# Child and adolescent mental well-being intervention programme: A systematic review of randomised controlled trials

**DOI:** 10.3389/fpsyt.2023.1106816

**Published:** 2023-04-06

**Authors:** Lawrence T. Lam, Mary K. Lam

**Affiliations:** ^1^Faculty of Medicine, Macau University of Science and Technology, Macau, Macao SAR, China; ^2^Faculty of Medicine and Health, The University of Sydney, Sydney, NSW, Australia; ^3^Faculty of Health, University of Technology Sydney, Sydney, NSW, Australia; ^4^RMIT University, Melbourne, VIC, Australia

**Keywords:** mental well-being, intervention, children, adolescents, randomised controlled trials, systematic review

## Abstract

**Background:**

There has been an increasing awareness and recognition of mental well-being as one of the main outcome measures in national mental health policy and service provision in recent years. Many systemic reviews on intervention programmes for mental health or general well-being in young people have been conducted; however, these reviews were not mental well-being specific.

**Objective:**

This study aims to examine the effectiveness of child and adolescent mental well-being intervention programmes and to identify the approach of effective intervention by reviewing the available Randomised Controlled Trials.

**Methods:**

This systematic review study followed the PRISMA guidelines for systematic reviews ensuring a methodical and structured approach for the literature search and the subsequent review processes. The systematic literature search utilised major medical and health databases. Covidence, an online application for conducting systematic reviews, was used to assemble the titles, abstracts and full articles retrieved from the initial literature search. To examine the quality of the included trials for determining the strength of the evidence provided, the JBI Critical Appraisal Tool for Randomised Controlled Trial was used.

**Results:**

There were 34 studies identified after an extensive search of the literature following the PRISMA guidelines. Seven (7) fulfilled all selection criteria and provided information on the effect of an intervention programme on mental well-being in adolescence. Data were extracted and analysed systematically with key information summarised. The results suggested that two (2) programmes demonstrated significant intervention effects, but with a small effect size. The quality of these trials was also assessed using the JBI Critical Appraisal Tool for Randomised Controlled Trials and identified some methodological issues.

**Conclusion:**

In conclusion, activity-based and psychoeducation are shown to be potentially effective approaches for future programme development. More research on a well-designed programme is urgently needed, particularly in developing countries, to provide good evidence in supporting the mental health policy through the enhancement of mental well-being in young people.

## Introduction

Positive mental health, as a concept representing self-acceptance, personal growth and actualisation, resilience, self-autonomy and mastery of the environment, has long been proposed (1). Instead of focusing on mental illness, there is an increasing emphasis on positive mental health and its effects on population health by the World Health Organization (2). Mental well-being has also been gaining much attention in the past two decades (2). The WHO defined positive mental health or good mental health as a: ‘state of well-being in which the individual realizes his or her own abilities, can cope with the normal stresses of life, can work productively and fruitfully, and is able to make a contribution to his or her community’ (3). This definition captures the concept that mental health is more than just an absence of mental illness (4). At the same time, there is also a growing acceptance that mental well-being, although closely resembles mental health, is a slightly different construct (5). Peterson has further defined mental well-being as: “the state of thriving in various areas of life, such as in relationships, at work, play, and more, despite ups and downs. It’s the knowledge that we are separate from our problems and the belief that we can handle those problems” (6). As the awareness and recognition of mental well-being have increased in recent years, it has become one of the main outcome measures in national mental health policy and service provision in many countries, particularly in the UK (7, 8).

In terms of the measurement of mental well-being, the concept encompasses multiple elements, so the construct is also complex (1). Assessment tools have been developed attempting to assess different aspects of mental well-being with some on the overall construct and others on specific domains. For example, the 5-item World Health Organization Well-being Index (WHO-5) was designed to assess the overall well-being of the mental state of an individual (9). The Mental Health Continuum-Short Form (MHC-SF) was another instrument developed for measuring three domains of well-being, namely emotional, psychological, and social (10). Based on the initial concept of mental well-being proposed by scholars in the field, such as Jahoda (1), Keyes (10), and Waterman (11), Tennant et al. proposed a two-dimensional model of mental well-being consisting of the hedonic and eudaimonic aspects (12, 13). The hedonic aspect refers to the individual subjective feeling of happiness and satisfaction in life, whereas the eudaimonic aspect is related to the psychological functioning and the actualisation of the individual’s potential, capacity, and positive relationship with self and others. Their efforts resulted in the development and validation of the Warwick–Edinburgh Mental Well-being Scale (WEMWBS) (13). A recent systematic review of the instruments for measuring mental wellness in adolescents suggested a range of core elements reflected from many different tools (14). Given the multiplicity of core elements embedded in the construct of mental well-being, it would be prudent to confine the selection of measuring instruments to those that include both hedonic and eudaimonic aspects, or the majority of items included in the instrument should cover these aspects.

As noted, there is a close relationship between mental well-being and mental health. This has been demonstrated in many studies (15–18). For example, in the cohort study on the effects of physical activity on mental well-being and mental health among adolescents aged 12–13 in England, Bell and colleagues found that there was a negative association between mental well-being scores, assessed by the WEMWBS, and scores of the Strength and Difficulties Questionnaire (*r* = −0.41) a measure of the mental health status (16). Another more recent study was conducted by Hides et al. on the relationship between mental well-being and psychological distress in a large sample of 2082 young people aged between 16 and 25 years in Australia. Results revealed that a bifactor model, in which mental well-being and distress were two separate constructs, was the only model that fitted well to the data with mental well-being and distress as subcomponents of mental health (18). While examining the relationship between changes in mental well-being and the inflammatory makers over time, Fancourt and Steptoe (17) discovered that elements of the two domains of mental well-being measures were negatively correlated to many inflammatory makers independent of the mental health status. These inflammatory markers had been identified to be associated with mental distress and ill health (17).

Mental Health problems among children and young adolescents have become a major public health issue. Global data indicated that the prevalence of mental health problems in children and adolescents was increasing a decade ago (19). Unfortunately, no improvement in the situation has been observed since then. On the contrary, the situation worsened in the past few years due to the COVID-19 pandemic (20). Early prevention of mental health problems is vital as mental health problems in almost half of adult patients start before the age of 14 (21). Good childhood mental health should be fostered during children’s early developmental processes. As mental well-being is an important aspect of good mental health, early intervention to promote mental well-being among children and adolescents is an important strategy for bettering mental health. If proven effective, this strategy will benefit not only young people but could potentially prevent mental ill health in the future adult population.

In terms of evidence-based practices, systematic reviews have been found on the intervention programmes for mental health or general well-being in young people; however, they were not mental well-being specific (22–26). While examining whether there are existing systematic reviews on the topic, main health-related databases were searched before the commencement of the current review study. The result is negative suggesting no previous review has been reported in the literature. In bridging the knowledge gap, this study aims, primarily, to examine the effect of child and adolescent mental well-being intervention programmes through a systematic review. It also attempts to identify the type of intervention programmes that have shown to be efficacious in bettering mental well-being in children and adolescents. To ensure the capturing of the best available evidence on the intervention programme, the review is limited to the reported Randomised Controlled Trials (RCT) only.

## Methods and materials

### Search strategies

This systematic review study followed the PRISMA guidelines for systematic reviews ensuring a methodical and structured approach for the literature search and the subsequent review processes (27). The systematic literature search utilised major medical and health databases including (1) PubMed, (2) ScienceDirect, (3) CINAHL full text, (4) AMED, (5) and MEDLINE.

In terms of the keywords and syntax used for the search, the following were used: (‘mental well-being OR mental wellbeing’) AND (intervention program OR intervention) AND (Randomised Controlled Trials). A slightly modified syntax was used per the requirements of the database. The following inclusion criteria were applied to the search: (1) the article was published in a peer-reviewed journal; (2) the article was written in the English language; (3) the study was an RCT of any type; (4) the outcome measure must fulfil the construct of mental well-being as defined above and (5) the target population of the RCT was children and adolescents. There was no restriction on the date of publication.

Covidence, an online application for conducting systematic reviews, was used to assemble the titles, abstracts, and full articles retrieved from the initial literature search. The steps below were undertaken to ensure all selection criteria of the review and the study selection for final data extraction, were satisfied. First, abstracts were screened for the required study type, and the trial was on an intervention programme for mental well-being in children and adolescents. Second, full texts of the selected articles from the previous step were examined to determine the suitability for data extraction. Both authors conducted the second step independently in accordance with the selection criteria. The results of the selection by the authors were then compared for similarities and to examine any discrepancies. Any differences in the selection were discussed and discrepancies were resolved by checking the selection criteria. Furthermore, to ensure that no other relevant studies might have been missed during the initial literature search, the reference lists of the selected articles for data extraction were also examined.

### Selection criteria

While selecting studies for data extraction, the following criteria were observed: (1) The study was an RCT with mental well-being as one of the main outcome variables; (2) The mental well-being of the participants was assessed using a validated instrument with the essential domains of the construct included; (3) Results on the effects of the intervention programme were clearly presented allowing for an estimate of the efficacy of the intervention and (4) The study was published in the English language.

### Information extraction, analysis, and publication quality assessment

**Figure 1:**
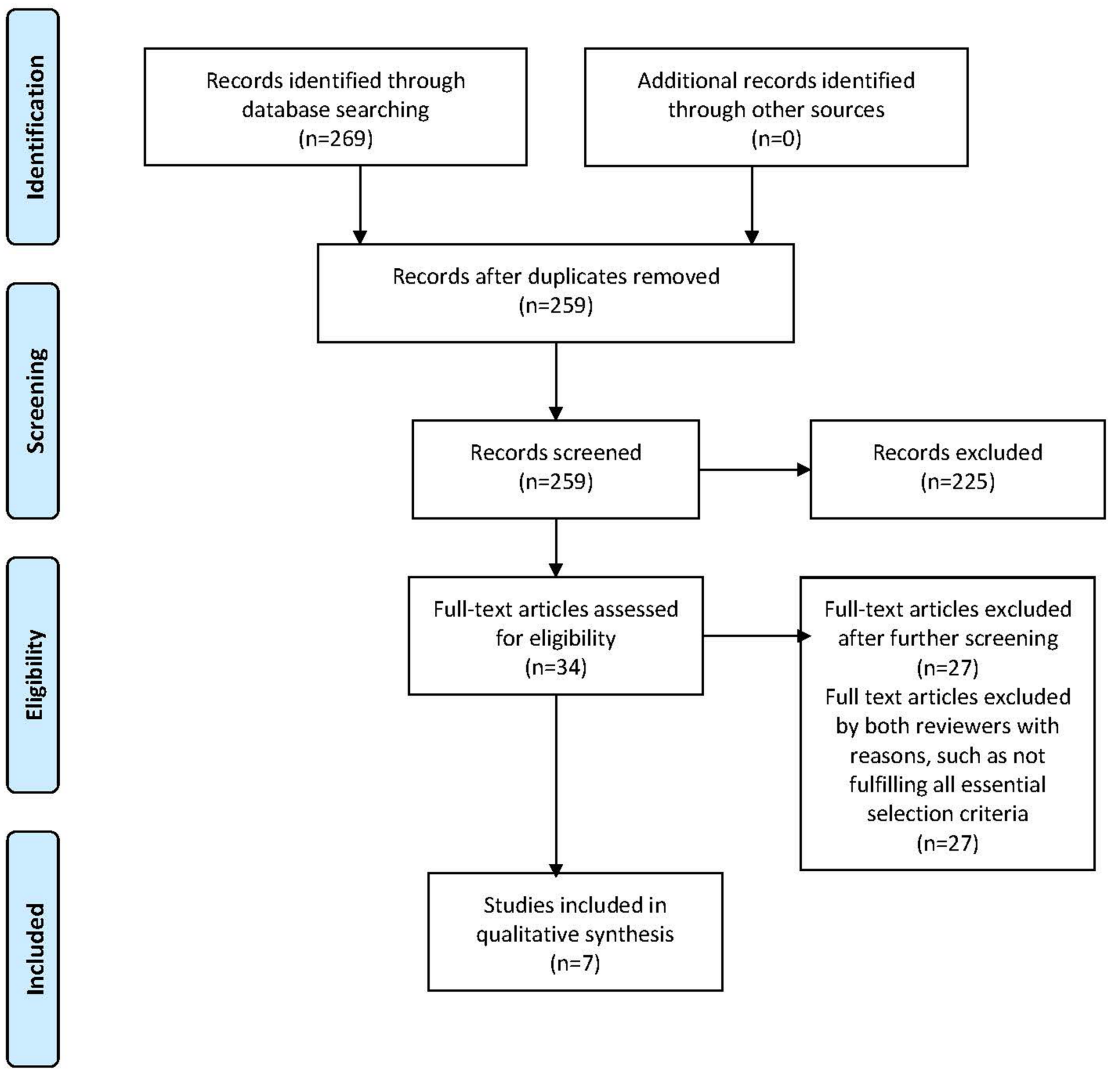
PRISMA flow chart.

For data extraction, information was captured from the included study and managed using the extraction tool provided in Covidence. This information included: authors, years of publication, location of the study, the study design, demographic characteristics of the sample, a description of the intervention programme, and the tools or instruments used to assess mental well-being. The results of the study, in terms of the effect of the intervention programme on mental well-being, were also recorded with an estimate of the effect size if available. The information was then summarised in a table for the analyses of a potential causal relationship between the intervention and the mental well-being of the participants. To examine the quality of the included trials for determining the strength of the evidence provided, the JBI Critical Appraisal Tool for Randomised Controlled Trial was used (28). The quality of each trial was rated against the JBI tool by both authors independently and then matched for similarities. Any discrepancies between the two were resolved by further reviewing the article for information. As the tool was not designed to be a psychometric scale, thus the assessment was conducted descriptively. Figure 1 depicts the PRISMA chart summarising the systematic literature searches and review process.

## Results

After following the literature search procedures on the five electronic databases, 34 articles were identified as potential studies for further screening. Of these 34 studies, only seven were found fulfilling all inclusion criteria (29–35). The main reasons for the exclusion of the 27 articles included: the outcome measure was not mental well-being as defined for this review study; the study design was not a proper RCT by the definition of a trial; the majority of the target population of the trial was not within the age range of children and adolescent. Data were extracted from the seven trials and information is summarised in Table 1. As shown, the sample size of these trials varied ranging from a small trial of 82 to the largest of 7,577 with a total of 10,357 participants aged younger than 19 years with two trials involving a small number of older young people (31, 35). In terms of the distribution of the sample size, two trials were large with more than a thousand participants, one medium size of about 500, and the rest were less than 200 (Table 1). The majority of these participants were recruited through schools or universities with some through social media and other communication means.

**Table 1:** Information extracted from the selected randomised controlled trials of intervention programmes for improving the mental well-being of children and adolescents.

Author, year, place (Reference)	Participants characteristics	Study design	Intervention programme and control condition	Outcome variable & measures	Confounding variables & measures	Method of analysis and adjustment for confounding	Results	Comments
Manicavasagar et al., 2014, Australia (34).	A total of 154 aged 12–18 years adolescents were recruited and completed the trial with 62 in the intervention group and 92 as controls. Participants were recruited through schools and youth organisation with the advertisement of the study using flyers.	A parallel two-arm randomised controlled trial	Intervention: Bite Back was an online positive psychology website utilising a combination of interactive exercises and information across 9 domains, including gratitude, optimism, flow, meaning, hope, mindfulness, character strengths, healthy lifestyle, and positive relationships. Control: two websites providing young viewers with news, comedy, drama, music, sports, and nature.	Mental Well-being was assessed by the short form of the Warwick–Edinburgh Mental Well-being Scale (WEMWBS-S)	No potential confounding variables were mentioned or adjusted in the analyses.	Date were analysed using 2-tailed Wilcoxon signed rank tests without adjustment for any confounding variables. However, there was no mention of the method of analysis for between-group comparisons.	There were no comparison results on the differences in mental well-being scores between the intervention and control groups postintervention. However, a significantly higher WEMEBS score was observed in the intervention group postintervention in comparison to the baseline (z = 2.07, *p* = 0.04).	This was a feasibility study of the acceptability of the online programme, thus no examination of the between group efficacy. Moreover, there was no sample size calculation. The sample might be sufficient for a feasibility study, but might not be sufficient for a full RCT.
Calear et al., 2016, Australia (29).	A total of 1767 high school students aged from 12 to 18 years with a mean age of 14.8 (s.d. = 0.97) with 37.2% males and 63.8% females completed the trial and data analysed. 562 and 427 were from the two intervention arms and 778 from the controls. Students were recruited from 32 schools within the vicinity of the 6 national Headspace centres.	A 3-arm cluster and stratified randomised controlled trial with the school as the randomisation unit. Students were nested in schools.	Intervention: An online e-couch Anxiety and Worry intervention programme (e-GAD) for generalised anxiety. The programme was an enhanced version of the original programme with the incorporation of access to a mental health service provider. The original e-GAD model involved education officers from the local Headspace centres supporting and assisting classroom teachers in delivering the programme. Controls: waitlist controls without mentioning any activities.	Mental well-being was assessed using the self-reported 14-item Warwick–Edinburgh Mental Well-being Scale (WEMWBS).	No potential confounding variables were mentioned for adjustment, although some between-groups differences were identified at baseline.	The Mixed Model with repeated measures (MMRM) were used for the analyses. The test for time and group-by-time effects was conducted.	There was a significant group-by-time interaction effect on Mental Well-being (*F* = 3.728, *p* = 0.001). *A priori* pair-wise comparisons resulted in significantly greater reductions in Mental Well-being for the intervention group at post-intervention (*t* = −2.1, *p* = 0.035) and at 6-month follow-up (*t* = −4.2, *p* < 0.001) for the eGAD School group in comparison to the controls. The e-GAD with health services had a significantly greater reduction in well-being at the 6-month follow-up (but not post-intervention) relative to the wait-list control condition (t = 3.3, *p* < 0.001).	A large-scale national study with a good representation of students from different backgrounds. The results on Mental Well-being were in the opposite direction against expectation, while other measures suggested a positive intervention effect.
Hides et al., 2019, Australia (31).	!69 participants, with 85 and 84 in the intervention and control groups respectively, were Australian residents aged 16 to 25 years, who reported at least mild distress in the past month on the Kessler 10 Psychological Distress scale (K10 > 17) and had an iPhone. They were recruited *via* student emails and posters in 2 large universities and snowballing techniques.	A waitlist randomised controlled trial	Intervention: The Music eScape app analyses each song in the users’ music library according to its level of valence (pleasant to unpleasant) and arousal (very low to very high) using The Echo Nest music data programme. The music choices were scanned to generate a mood map for the user. The app will prompt the user to reflect his/her current mood and encourage plotting a mood journey. User will be asked to reflect on their mood upon the completion of the playlist. Control: the waitlist group received 2 SMS text messages during the 1 month wait for access to the app.	Mental well-being was measured with the Mental Health Continuum-Short Form (MHC-SF)	Gender, duration of music use, the use of music, app access, and app use were included as potential confounding variables.	Data were analysed using Linear Mixed Models with intention-to-treat analyses. Time and time-by-group interaction effect analyses were conducted.	There was no significant time-by-group effect for mental well-being, but a significant time effect that was not moderated by any other variables. A significant time effect was found when comparing the assessment at 3-month to baseline (mean diff = 3.09, 95% CI = 0.8805.29, t278 = 2.76, *p* = 0.006, d = 0.33).	A small-scale trial with only two universities as the sampling frame. The snowballing method of recruitment might incur some sampling biases. The sample consisted of participants outside of the targeted population.
Dowling et al., 2019, Ireland (30).	A total of 497 high school students aged between 15 and 18 years from 32 schools were identified as designated disadvantaged status by the Department of Education and Skills of Ireland. Of these, 246 were allocated to the intervention group and 251 controls with a nearly equal number of males and females in both groups. Schools were recruited through the list of Disadvantaged Schools registered with the Department of Education of Ireland.	A stratified cluster randomised controlled trial with schools as the randomisation unit. Students were nested in schools.	Intervention: The MindOut programme is a 13-week school-based programme incorporated into the Social and Personal Health Education curriculum. The programme consists of five core components for social and emotional learning including self-awareness, self-management, social awareness, relationship management, and responsible decision-making. Controls: the waitlist group with Teaching as Usual (TAU).	Mental well-being was assessed using the self-reported 14-item Warwick–Edinburgh Mental Well-being Scale (WEMWBS).	Gender and the baseline assessment score were included in the analyses as covariates.	To cater for the clustering effect of the sample, Linear Mixed Model (LMM) were applied with intention-to-treat analyses.	No significant intervention effects on self-reported mental well-being were found (*p* = 0.942)	A medium-sized trial with a reasonable study design.
Kuroko et al., 2020, New Zealand (32)	A total of 111 adolescents aged 12–15 years completed the baseline and the 7-week post-intervention assessments (85 in the intervention, 26 in the control groups) with 113 at 12-month follow-up (86 in the intervention, 27 in the control groups). Participants were recruited through social media, posters, and word-of-mouth.	A parallel two-arm randomised controlled trial	Intervention: The adolescent cooking intervention programme was a school-based holiday activity. Young people received an intensive 5-day practical cooking programme at school. After that, they received a home-based, social media-led 6-week home cooking with a weekly meal kit provided. Controls: received no active activities only the completion of the study measures.	Mental Well-being was assessed using the 5-item World Health Organization Well-being Index (WHO-5).	No confounding variables were mentioned and adjusted in the analyses.	Mixed Regression Models with intention-to-treat were used for data analyses. An interaction term between time and group was included in the model with participants and group as the random effect.	There was a significant difference in the change in mean mental well-being scores from baseline to 7 weeks with a mean difference score of 3 (p = 0.005) in favour of the intervention group. However, no difference was found between groups at the 12-month follow-up.	A small-sized trial with the sample recruited from a city. The method of recruitment might incur some sampling biases.
Thabrew et al., 2022, New Zealand (35).	A total of 82 young people aged 16–30 years, with a mean age of 23 years and the majority were females (more than 80%), were recruited through social media and completed the trial.	A parallel 2-arm randomised controlled trial	Intervention: A mobile app specifically designed for the trial and downloaded from the App Store. It consisted of seven positive psychology, CBT, and psychoeducation-based modules that would be completed within 7 days. Control: the waitlist group with no specific activities mentioned.	Mental Well-being was assessed using the 5-item WHO Well-Being Index (WHO-5)	Not mentioned although demographic and health information was collected.	Linear Mixed models with the inclusion of group and time interaction effects were applied for the comparison of group means. *Post hoc* tests were used to assess pairwise comparisons of the group at each time point and within-group changes.	Results indicated a significant time-by-group interaction effect on Mental Well-being (*p* = 0.043). Significant differences in the mean score of Mental Well-being were found between groups in favour of the intervention with 13.19 (9% C.I. 3.96–22.42) at 4 weeks and 13.77 (95% CI = 4.50–23.03) at 3 months with an overall effect size of Cohen’s f2 = 0.05.	Power calculation was conducted for a sample size of 90 to provide a study power of 90%. With the final sample of 82, the study should retain a good level of power for the conclusion.
Kuyken et al., 2022, UK (33).	A total of 7,577 secondary school students, with an average age of 12.2 years (s.d. = 0.6) from 84 schools in the UK with 3,779 and 3,798 in the intervention and control groups at post-intervention, respectively. Of these, 3,678 and 3,572 students remained in the intervention and control groups at 12 months follow-up. Schools were recruited to the trial as a national project.	A cluster randomised control trial with schools as the unit of randomisation.	Intervention: School-based mindfulness training designed to address a broad spectrum of youth mental health issues. Control: Teaching as usual (TAU)	Mental well-being was assessed using the 14-item self-reported Warwick Edinburgh Mental Well-being Scale (WEMWBS).	The outcomes were adjusted for the factors used to balance randomisation, cohort, student gender and baseline score on the outcome.	The mixed effect linear regression models were used for data analyses with a test of the interaction effect between time and intervention group.	There was no evidence of a significant intervention effect on mental well-being or an interaction effect with a very small effect size of 0.02 (95% CI = −0.03 to 0.07).	A large-scale trial with a representative sample.

For the study design, of the seven RCTs three were parallel arms trials on individual participants (32, 34, 35), three were cluster randomised controlled trials, with or without stratification (29, 30, 33), and one randomised wait-listed control trial (31). In terms of dates of the studies, most of these were recent studies with five being conducted within the past 5 years. All trials were implemented in developed countries with three in Australia (29, 31, 34)), two in New Zealand (32, 35), one in Ireland (30), and one in the UK (33). All studies utilised a standardised self-reported instrument for the assessment of mental well-being at the baseline and post-intervention. Four trials utilised the WEMWBS (29, 30, 33, 34), two used the WHO-5 (32, 35), and Hides et al. (31) employed the MHC-SF as the assessment tool.

In terms of intervention programmes, nearly half (*n* = 3, 43%) were using a psychoeducation approach, either school-based, online or App-based (30, 34, 35). Two were trials on e-couching methods of positive psychological training with one utilising additional face-to-face services and the other using an App-based musical mood training programme (13, 29). One study applied an individualised activity-based approach of a cooking programme (32), and one was a school-based mindfulness programme (33).

The efficacy of these intervention programmes was also analysed. Of the seven trials, only two demonstrated a significant effect of the intervention programme with both being conducted in New Zealand. Kuroko’s cooking intervention programme resulted in a significant difference in the change in mean mental well-being scores from baseline to 7 weeks with a mean difference score of 3 (*p* = 0.005) in favour of the intervention group (32). The psychoeducation programme conducted by Thabrew et al. (35) also found significant differences in the mean score of mental well-being between groups in favour of the intervention with 13.19 (9% CI 3.96–22.42) at 4 weeks and 13.77 (95% CI = 4.50–23.3) at 3 months with an overall small effect size of Cohen’s *f*^2^ = 0.05. The other trials found no significant intervention effects. One did not conduct comparisons between groups.

The quality of these studies was also assessed with the application of the JBI Critical Appraisal Tool for Randomised Controlled Trials. The results of the assessment are summarised in Table 2. As noted, most of these trials were of acceptable quality with many of the items scoring positive. However, owing to the study design of these trials with the use of online programme delivery and data collection, some of the items were unavailable for assessment. Particularly, items related to the blinding of treatment assignment to the participants, to the treatment deliverers, and to the assessors of outcomes. Another item of concern was related to the treatment applied to different arms of the trial at baseline. Most of the reports did not provide sufficient information for the assessment of this item. Furthermore, the follow-up of participants, either for post-intervention assessment or for longer-term assessments, was unclear in many of the trials. More detailed analyses of these reports showed that of these seven trials more than half (n = 4, 57%) were small-sized and might not be able to provide sufficient power for the study (Table 1). Moreover, one trial did not conduct a comparison of the outcome between groups (34). On the whole, the quality of these trials improved over time.

**Table 2:** Results on the assessment of the quality of the selected studies.

Items	Studies						
	Manicavasagar et al.	Calear et al.	Hides et al.	Dowling et al.	Kuroko et al.	Thabrew et al.	Kuyken et al.
Was true randomisation used for the assignment of participants to treatment groups?	NA	✔	✔	✔	✔	✔	✔
Was allocation to treatment groups concealed?	✔	✔	✔	NA	NA	NA	✔
Were treatment groups similar at the baseline?	NA	✖	✔	?	✖	?	✔
Were participants blind to treatment assignment?	NA	NA	NA	NA	NA	NA	NA
Were those delivering treatment blind to treatment assignment?	NA	✖	NA	NA	NA	NA	NA
Were outcomes assessors blind to treatment assignment?	?	✔	NA	?	?	NA	?
Were treatment groups treated identically other than the intervention of interest?	NA	✔	✔	✔	✔	✔	✔
Was follow-up complete and if not, were differences between groups in terms of their follow-up adequately described and analysed?	?	?	✖	?	✔	✔	?
Were participants analysed in the groups to which they were randomised? (ITT)	✖	✔	✔	✔	✔	✔	✔
Were outcomes measured in the same way for treatment groups?	NA	✔	✔	✔	✔	✔	✔
Were outcomes measured in a reliable way?	✔	✔	✔	✔	✔	✔	✔
Was appropriate statistical analysis used?	✔	✔	✔	✔	✔	✔	✔
Was the trial design appropriate, and were any deviations from the standard RCT design (individual randomisation, parallel groups) accounted for in the conduct and analysis of the trial?	?	✔	✔	✔	✔	✔	✔

✔, Yes; ✖, No;?, Unclear; NA, Not available due to study design.

## Discussions and conclusion

There are two aims of this study. First, to examine the possible effects of different intervention programmes on the mental well-being of children and adolescents through a systematic review of Randomised Controlled Trials. Second, to identify the type of intervention programmes, particularly the main contents that are shown to be efficacious for improving the mental well-being of young people. The results of the review suggest that not many well-designed RCTs were conducted in the past. The more recent studies carried out in the last 5 years were of better quality. Among the seven reviewed trials, only two demonstrated a significant effect of the implemented intervention on the mental well-being of participants. However, these two New Zealand trials were both of smaller size with one having 111 and the other 82. In terms of the effect of the intervention, while one study reported small effect size, the information provided in the articles was not sufficient to conduct a proper calculation on the treatment effect in comparison to the smallest worthwhile effect (36). In terms of the contents of the intervention programmes, one was activity-based, and the other was education-based programmes. Given the lack of a systematic review of a similar topic, this study would be considered unique and the first in the area.

The results obtained from this review provided some insights into the current development of intervention programmes for the advancement of mental good health *via* the improvement of mental well-being, particularly among children and young adolescents. As aforementioned in the introduction, mental well-being has become an important outcome measure in national mental health policy and service provision in many countries, including the UK (8). For example, based on the framework and the agenda of the WHO Comprehensive Mental Health Action Plan (37), the European Mental Health Action Plan was formulated with the first main objective: ‘Everyone has an equal opportunity to realize mental well-being throughout their lifespan, particularly those who are most vulnerable or at risk’ (38). Given the recognition and the strong advocacy for mental well-being as an important element in the overall strategy of mental health, it is surprising to see that there have not been many well-designed intervention programmes validated by strong research methodologies and implemented as shown in this systematic review. As such, there is an urgent need to further research into the development and validation of high-quality intervention programmes for enhancing mental well-being among young people. Drawing upon the existing evidence provided by this review, activity-based and psychoeducation intervention would be a reasonable approach for the consideration of future programme development.

There are strengths and limitations in all studies, and so do in this systematic review. The PRISMA guidelines for systematic reviews were followed closely to ensure the study’s validity and scientific rigour. Both reviewers observed the criteria for article selection and the procedures stipulated by the guidelines reaffirming the standards of the reviewing processes minimising the selection bias. The employment of the online platform Covidence reduced operational errors and provided a standard approach to data extraction and summarising the extracted information. For the limitations on individual studies, comments were provided in the summary table. Readers can refer to Table 1. Some limitations have been identified in this review study. First, there were too few studies on the topic for conducting a meta-analysis on the effect of the intervention programmes. Second, the sample sizes of most of the included studies were rather small resulting in the possibility in lacking study power to demonstrate a true effect should there be one. Third, in terms of the outcome measure, these trials utilised three different instruments with the WEMWBS being the most common. Although all instrument attempt to assess the construct of mental well-being, there are still some differences among them. This might, in some way, introduce some assessment biases to the study and would possibly explain the differences in the results obtained in different trials. It is recommended that, as far as possible, a standard instrument with the best psychometric properties should be used for future studies.

The current review study has some important contributions to the field of public mental health. Theoretically, the concepts of mental health and mental well-being have been clearly defined and distinguished in this study. The differences between these two concepts should be highlighted for researchers in the field so that scientific pursuits in the understanding of the risk and protective factors of these mental states could be better achieved. In terms of the practical significance, the results of this review have provided some pointers for practitioners in the field in designing future intervention programmes for the enhancement of the mental well-being in young people. In general, programmes adopting a multiple approach of psychoeducation and activities with the employment of the latest communication technologies would be more effective.

In conclusion, this systematic review has examined the available trials on the effect of different intervention programmes on mental well-being among children and adolescents. The results suggest that psychoeducation for positive mental health and psychological well-being and activity-based programme might be effective approaches for intervention. More research on a well-designed programme is urgently needed, particularly in developing countries, to provide good evidence in supporting the mental health policy through the enhancement of mental well-being in young people.

## Data availability statement

The original contributions presented in the study are included in the article/supplementary material, further inquiries can be directed to the corresponding author.

## Author contributions

LL and ML were involved in the design of the review, literature search, screening of articles, selection of studies to be reviewed, data extraction, summarizing the information, and drafting and reviewing the manuscript. All authors read and approved the final manuscript. The allocation of authorship is in accordance with the International Committee of Medical Journal Editors (ICMJE) requirements.

## Conflict of interest

The authors declare that in the conduct of research and the production of the manuscript there are no commercial or financial relationships that could be considered a potential conflict of interest.

## Publisher’s note

All claims expressed in this article are solely those of the authors and do not necessarily represent those of their affiliated organizations, or those of the publisher, the editors and the reviewers. Any product that may be evaluated in this article, or claim that may be made by its manufacturer, is not guaranteed or endorsed by the publisher.

## References

[1] Jahoda M. Current concepts of positive mental health. New York: Basic Books (1958) doi: 10.1037/11258-000

[2] WHO. World mental health report: transforming mental health for all. Licence: CC BY-NC-SA 3.0 IGO. Geneva: World Health Organization (2022).

[3] WHO. Promoting mental health: Concepts, emerging evidence, practice (summary report). Geneva: World Health Organization (2004).

[4] Stewart-Brown S Samaraweera PC Taggart F Kandala NB Stranges S. Socioeconomic gradients and mental health: implications for public health. Br J Psychiatry. (2015) 206:461–5. doi: 10.1192/bjp.bp.114.147280, PMID: 25792696

[5] Haworth CM Carter K Eley TC Plomin R. Understanding the genetic and environmental specificity and overlap between well-being and internalizing symptoms in adolescence. Dev Sci. (2017) 20:e12376. doi: 10.1111/desc.12376, PMID: 26709037 PMC5347864

[6] Peterson T. (2021). What is mental wellbeing? Definition and examples, HealthyPlace. Available at https://www.healthyplace.com/self-help/self-help-information/what-mental-wellbeing-definition-and-examples. (Accessed March 10, 2023).

[7] de Cates A Stranges S Blake A Weich S. Mental well-being: an important outcome for mental health services? Br J Psychiatry. (2015) 207:195–7. doi: 10.1192/bjp.bp.114.158329 26329562

[8] Garratt K Laing J. Mental health policy in England. London: House of Parliament Library (2022).

[9] WHO. Wellbeing measures in primary health care/the Depcare project. Copenhagen: WHO Regional Office for Europe (1998).

[10] Keyes CL. Mental illness and/or mental health? Investigating axioms of the complete state model of health. J Consult Clin Psychol. (2005) 73:539–48. doi: 10.1037/0022-006X.73.3.539 15982151

[11] Waterman AS. Two conceptions of happiness: contrasts of personal expressiveness (eudaimonia) and hedonic enjoyment. J Pers Soc Psychol. (1993) 64:678–91. doi: 10.1037/0022-3514.64.4.678

[12] Joshanloo M Weijers D. A two-dimensional conceptual framework for understanding mental well-being. PLoS One. (2019) 14:e0214045. doi: 10.1371/journal.pone.0214045, PMID: 30917191 PMC6436799

[13] Tennant R Hiller L Fishwick R Platt S Joseph S Weich S. The Warwick-Edinburgh mental well-being scale (WEMWBS): development and UK validation. Health Qual Life Outcomes. (2007) 5:63. doi: 10.1186/1477-7525-5-63, PMID: 18042300 PMC2222612

[14] Orth Z Moosajee F Van Wyk B. Measuring mental wellness of adolescents: a systematic review of instruments. Front Psychol. (2022) 13:835601. doi: 10.3389/fpsyg.2022.835601, PMID: 35356328 PMC8959676

[15] Purba A Demou E. The relationship between organisational stressors and mental wellbeing within police officers: a systematic review. BMC Public Health. (2019) 19:1286. doi: 10.1186/s12889-019-7609-0, PMID: 31615479 PMC6792329

[16] Bell SL Audrey S Gunnell D Cooper A Campbell R. The relationship between physical activity, mental wellbeing and symptoms of mental health disorder in adolescents: a cohort study. Int J Behav Nutr Phys Act. (2019) 16:138. doi: 10.1186/s12966-019-0901-7, PMID: 31878935 PMC6933715

[17] Fancourt D Steptoe A. The longitudinal relationship between changes in wellbeing and inflammatory markers: are associations independent of depression? Brain Behav Immun. (2020) 83:146–52. doi: 10.1016/j.bbi.2019.10.004, PMID: 31604140 PMC6928572

[18] Hides L Quinn C Stoyanov S Cockshaw W Kavanagh DJ Shochet I. Testing the interrelationship between mental well-being and mental distress in young people. J Posit Psychol. (2020) 15:314–24. doi: 10.1080/17439760.2019.1610478

[19] WHO. Adolescent mental health: Mapping actions of nongovernmental organization and other international development organization. Geneva: World Health Organisation (2012).

[20] Kauhanen L Wan Mohd Yunus WMA Lempinen L Peltonen K Gyllenberg D Mishina K. A systematic review of the mental health changes of children and young people before and during the COVID-19 pandemic. Eur Child Adolesc Psychiatry. (2022) 12:1–19. doi: 10.1007/s00787-022-02060-0, PMID: 35962147 PMC9373888

[21] WHO. (2019). *Mental health*. Available at: https://www.who.int/mental_health/maternal-child/child_adolescent/en/. (Accessed September 31, 2022).

[22] Barry MM Clarke AM Jenkins R Patel V. A systematic review of the effectiveness of mental health promotion interventions for young people in low and middle income countries. BMC Public Health. (2013) 13:835. doi: 10.1186/1471-2458-13-835, PMID: 24025155 PMC3848687

[23] Cahill SM Egan BE Seber J. Activity- and occupation-based interventions to support mental health, positive behavior, and social participation for children and youth: a systematic review. Am J Occup Ther. (2020) 74:7402180020p1-7402180020p28. doi: 10.5014/ajot.2020.038687, PMID: 32204773

[24] Clarke AM Kuosmanen T Barry MM. A systematic review of online youth mental health promotion and prevention interventions. J Youth Adolesc. (2015) 44:90–113. doi: 10.1007/s10964-014-0165-0, PMID: 25115460

[25] Fenwick-Smith A Dahlberg EE Thompson SC. Systematic review of resilience-enhancing, universal, primary school-based mental health promotion programs. BMC Psychology. (2018) 6:30. doi: 10.1186/s40359-018-0242-3, PMID: 29976252 PMC6034212

[26] Schmidt M Werbrouck A Verhaeghe N Putman K Simoens S Annemans L. Universal mental health interventions for children and adolescents: a systematic review of health economic evaluations. Appl Health Econ Health Policy. (2020) 18:155–75. doi: 10.1007/s40258-019-00524-0, PMID: 31605299

[27] Moher D Liberati A Tetzlaff J Altman DG for the PRISMA Group. Preferred reporting items for systematic reviews and meta-analyses: the PRISMA statement. Br Med J. (2009) 339:b2535–336. doi: 10.1136/bmj.b2535 21603045 PMC3090117

[28] Tufanaru C Munn Z Aromataris E Campbell J Hopp L. Systematic reviews of effectiveness In: Aromataris E Munn Z, editors. JBI manual for evidence synthesis. London, UK: JBI (2020).

[29] Calear AL Batterham PJ Poyser CT Mackinnon AJ Griffiths KM Christensen H. Cluster randomised controlled trial of the e-couch anxiety and worry program in schools. J Affect Disord. (2016) 196:210–7. doi: 10.1016/j.jad.2016.02.049, PMID: 26926660

[30] Dowling K Simpkin AJ Barry MM. A cluster randomized-controlled trial of the MindOut social and emotional learning program for disadvantaged post-primary school students. J Youth Adolesc. (2019) 48:1245–63. doi: 10.1007/s10964-019-00987-3, PMID: 31004264

[31] Hides L Dingle G Quinn C Stoyanov SR Zelenko O Tjondronegoro D. Efficacy and outcomes of a music-based emotion regulation mobile app in distressed young people: randomized controlled trial. JMIR Mhealth Uhealth. (2019, 2019) 7:e11482. doi: 10.2196/11482 30664457 PMC6352004

[32] Kuroko S Black K Chryssidis T Finigan R Hann C Haszard J. Create our own Kai: a randomised control trial of a cooking intervention with group interview insights into adolescent cooking Behaviours. Nutrients. (2020) 12:796. doi: 10.3390/nu12030796, PMID: 32197342 PMC7146447

[33] Kuyken W Ball S Crane C Ganguli P Jones B Montero-Marin J. Effectiveness and cost-effectiveness of universal school-based mindfulness training compared with normal school provision in reducing risk of mental health problems and promoting well-being in adolescence: the MYRIAD cluster randomised controlled trial. Evid Based Ment Health. (2022) 25:99–109. doi: 10.1136/ebmental-2021-300396, PMID: 35820992 PMC9340028

[34] Manicavasagar V Horswood D Burckhardt R Lum A Hadzi-Pavlovic D Parker G. Feasibility and effectiveness of a web-based positive psychology program for youth mental health: randomized controlled trial. J Med Internet Res. (2014) 16:e140. doi: 10.2196/jmir.3176, PMID: 24901900 PMC4071231

[35] Thabrew H Boggiss AL Lim D Schache K Morunga E Cao N. Well-being app to support young people during the COVID-19 pandemic: randomised controlled trial. Br Med J Open. (2022) 12:e058144. doi: 10.1136/bmjopen-2021-058144, PMID: 35589362 PMC9121135

[36] Ferreira ML Herbert RD Ferreira PH Latimer J Ostelo RW Nascimento DP. A critical review of methods used to determine the smallest worthwhile effect of interventions for low back pain. J Clin Epidemiol. (2012) 65:253–61. doi: 10.1016/j.jclinepi.2011.06.018, PMID: 22014888

[37] WHO. Comprehensive mental health action plan 2013–2020. WHA66.8. Geneva: World Health Organization (2013).

[38] WHO. The European mental health action plan 2013–2020. Geneva: World Health Organization (2015).

